# Digital Image Correlation for Measuring Full-Field Residual Stresses in Wire and Arc Additive Manufactured Components

**DOI:** 10.3390/ma16041702

**Published:** 2023-02-17

**Authors:** Dibakor Boruah, Nele Dewagtere, Bilal Ahmad, Rafael Nunes, Jeroen Tacq, Xiang Zhang, Hua Guo, Wim Verlinde, Wim De Waele

**Affiliations:** 1 Department of Electromechanical, Systems and Metal Engineering, Faculty of Engineering and Architecture, Ghent University, 9052 Ghent, Belgium; 2 Faculty of Engineering, Environment and Computing, Coventry University, Coventry CV1 5FB, UK; 3 Belgian Welding Institute, 9052 Ghent, Belgium; 4 Sirris, 9052 Ghent, Belgium

**Keywords:** additive manufacturing, contour method, digital image correlation, residual stresses, wire + arc additive manufacturing, X-ray diffraction

## Abstract

This study aims to demonstrate the capability of the digital image correlation (DIC) technique for evaluating full-field residual stresses in wire and arc additive manufactured (WAAM) components. Investigations were carried out on WAAM steel parts (wall deposited on a substrate) with two different wall heights: 24 mm and 48 mm. Mild steel solid wire AWS ER70S-6 was used to print WAAM walls on substrates that were rigidly clamped to H-profiles. DIC was used to monitor the bending deformation of WAAM parts during unclamping from the H-profiles, and residual stresses were calculated from the strain field captured during unclamping. Residual stresses determined from the proposed DIC-based method were verified with an analytical model and validated by the results from established residual stress measurement techniques, i.e., the contour method and X-ray diffraction.

## 1. Introduction

Wire and arc additive manufacturing (WAAM) is one among various emerging metal additive manufacturing (AM) processes that fall under the umbrella term directed energy deposition (DED). In WAAM, a metal wire is fed at a controlled rate into an electric arc to melt the filler metal onto previously deposited layers or a substrate, allowing the manufacture of multi-material products. WAAM offers high material usage efficiency and low cost, as well as high deposition speed (typically 1–10 kg/h depending on the material), revealing the potential to fabricate large custom-made metal parts with increased design freedom (up to 1 mm resolution) [1,2]. WAAM has attracted a lot of attention to fabricating components using various steel grades as well as non-ferrous alloys. However, a persistent challenge associated with WAAM is the process-induced residual stress and distortion or deformation of the printed parts. Localised heating and uneven cooling during the WAAM process introduce large thermal gradients causing distortion and residual stresses, which can affect the topology and global integrity of a WAAM component [3]. In the last decade, WAAM induced residual stresses have been experimentally investigated for various alloys, such as steel [4,5,6,7,8,9,10,11,12], aluminium [13,14,15,16,17,18,19,20], titanium [3,13,21,22,23,24,25,26,27,28,29,30], nickel [13,29,31,32,33], intermetallics [21,34,35], etc. The aforementioned literature has focused on quite a few areas including but not limited to the effect of process and geometrical variables on residual stresses, the effect of interpass and side rolling on controlling/reducing residual stresses, and the effect of pre-and post-processing on residual stresses, etc. Some of the commonly used experimental methods for measuring residual stresses in WAAM parts are neutron diffraction [36], X-ray diffraction (XRD) [37], contour method [38], and incremental centre hole drilling [39].

There are several methods for residual stress evaluation, each having its advantages and limitations, with the majority of them only capable of measuring local residual stresses, as presented in Table 1. Non-destructive methods that are highly reliable and widely accepted such as neutron diffraction have only a few facilities across the globe, which are highly expensive with limited accessibility. The contour method is another popular technique that has the advantage of having no limitation on specimen thickness; however, it is destructive, expensive, and can provide measurement only in one direction. In the recent past, there has been a growing interest in digital measurements of deformation and strain. Full-field digital image correlation (DIC) is an optical non-contact technique that allows quantification of displacement and deformation in multiple directions, covering a large range of length scales (microns to meters) [40]. Aside from the initial investment, DIC requires almost no maintenance and no consumables. Moreover, commonly used experimental methods (as listed on Table 1) do not provide any information on the deformation history, unlike the DIC technique. Previously, DIC has been used to quantify residual stresses in various applications; some are completely performed in a non-destructive way [41,42,43,44] and some are combined with a destructive measurement technique such as DIC-aided hole drilling [45,46,47,48], DIC-aided slitting [40,49], DIC-aided focused ion beam (FIB) micro ring/region milling/drilling [50,51,52], etc. Application of DIC for residual stress measurements includes composites [40], selective laser-melted parts [43,51], arc-welded steel tubes [41] and plates [44], friction stir butt-welded joints [49], thin-film coatings [42], cold spray deposits [52], plasma spray coatings [53], etc. A review article by Cunha et al. [54] provided a comprehensive overview of the in situ monitoring of additive manufacturing using DIC. A case study on in situ monitoring of WAAM SS 316L parts using DIC and associated challenges was also reported in [54]. However, according to the authors’ best knowledge, the applicability of DIC for measuring full-field residual stresses in WAAM components has not yet been explored.

In this study, a DIC-based methodology was developed to evaluate full-field residual stresses in WAAM parts. Steel alloy AWS ER70S-6 was selected as a feedstock wire to build WAAM walls with two different heights: 24 mm and 48 mm. Further, DIC measured residual stresses were validated using the contour method and XRD as well as an analytical model.

## 2. Experimental Methods

### 2.1. Feedstock Wire and Substrate Material

A commercially available mild steel solid wire AWS A5.18 ER70S-6 (EN ISO 14341-A: G 46 4 M21 3Si1/G 42 3 C1 3Si1) with 1 mm diameter (MicroguardTM Ultra) supplied by Lincoln Electric (Nijmegen, Netherlands) was used as the consumable electrode to produce all specimens. For the substrate, carbon steel S355 with a nominal size 200 mm × 100 mm × 10 mm was used. The chemical composition of the wire and the substrate material are shown in Table 2. Mechanical properties according to the wire supplier datasheet are: yield strength 502 MPa, tensile strength 574 MPa, elongation 28%, and impact toughness at 40 °C is 102 J.

### 2.2. WAAM Process and Specimen Preparation

All specimens produced for the study were printed by the Belgian Welding Institute (BWI). The WAAM equipment comprises a KUKA welding robot for the gas metal arc welding (GMAW) process and a Fronius cold metal transfer (CMT) welding source. Key process parameters are presented in Table 3.

**Figure 1:**
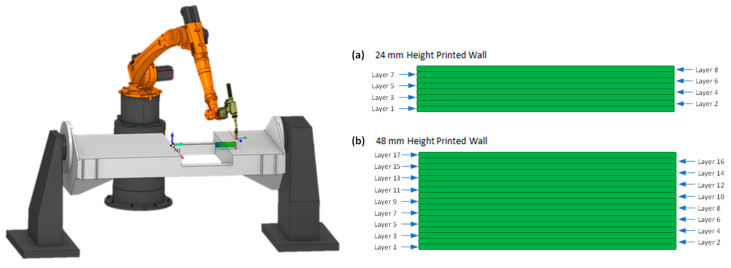
Robotic WAAM cell with the zig-zag deposition path strategy used in the printing for the walls with (**a**) 24 mm height, and (**b**) 48 mm height.

As already well discussed in the literature [3,33,56], the WAAM deposition path strategy has a significant influence on the heat concentration and, therefore, thermal deformation and residual stress developed in the component. With this in mind, the deposition path strategy “Zig-Zag” was kept fixed for all specimens. This deposition path strategy was elaborated with the SprutCam 14 software (Figure 1).

To study the feasibility of DIC as a residual stress measurement technique for WAAM, an investigation was carried out for “wall” specimens with two different heights: (a) 24 mm and (b) 48 mm. For each type, two specimens were used to study repeatability in DIC measurements.

**Figure 2:**
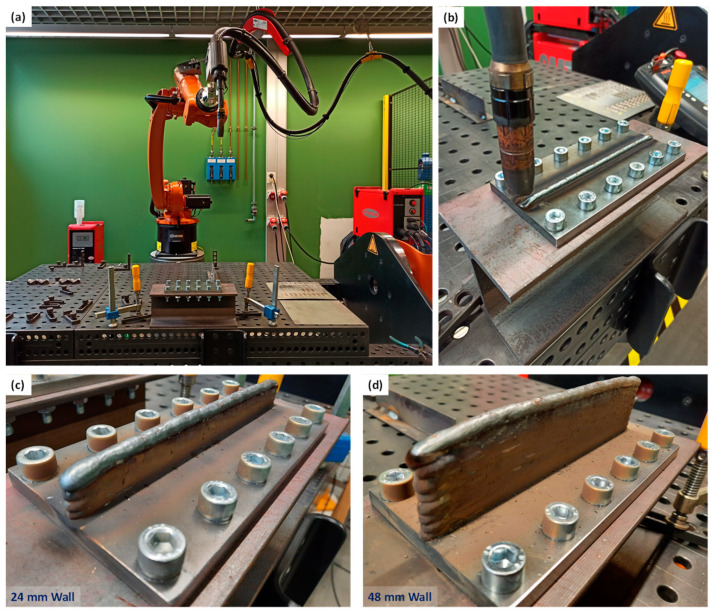
(**a**) WAAM set-up at the Belgian Welding Institute, (**b**) a closer view showing the printing process (after deposition of a single bead layer) on a substrate clamped on an H-profile, which is clamped on the table. Printed wall specimens with two different heights: (**c**) 24 mm, and (**d**) 48 mm.

Before printing, substrates were clamped rigidly to H profiles that were clamped onto a rigid table above which the robotic arm could manoeuvre. The idea was to keep close-to-zero deformation in the substrate during the deposition process, and only release them in a controlled manner when the component was monitored using DIC. For this purpose, the substrate should be clamped on a portable, rigid structure. An H-or I-profile appeared to be suitable for serving as the support structure for the substrate, as this made it easier to reach for the bolts during (dis)assembly, prevented any bending moments and still provided a lightweight, rigid solution. An H-profile was preferred over an I-profile, as there was more room for the bolts and the unscrewing process. A standard H profile, HE 120 A (cut to 300 mm length) was chosen, made of steel grade S275. The clamping of the substrate was accomplished by bolting, as this provided high rigidity and a uniform force distribution. The fastening was accomplished with M10 socket bolts (grade 8.8), washers, and nuts, using 12 bolts for each substrate. A manual torque wrench and key were used to achieve equal clamping conditions for all bolts, and the torque being applied was ~38 Nm. The H-profile and substrate configuration with the WAAM printing set-up at BWI are shown in Figure 2a–d present two printed wall specimens with 24 mm and 48 mm height. More details on the specimens used in this study are listed in Table 4.

### 2.3. Residual Stress Evaluation Using Digital Image Correlation

#### 2.3.1. The Concept

**Figure 3:**
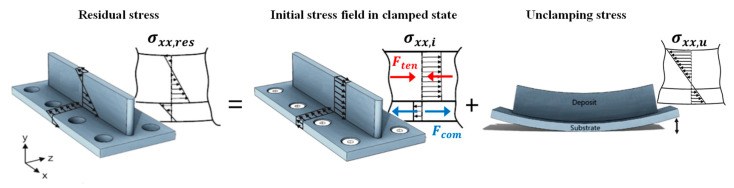
Schematic representation of longitudinal residual stress being the summation of the initial stress field in clamped state and unclamping stress (based on [5,13,57,58]). F_ten_ and F_com_ represent the tensile force in the printed wall and the compressive force in the substrate, respectively.

Figure 3 illustrates the distribution of the longitudinal component of residual stress $\left(\sigma_{xx,\;res}\left(y\right)\right)$ in a WAAM part. If the substrate is clamped rigidly during printing, the residual stress is the summation of the initial stress field before unclamping $\left(\sigma_{xx,\;i}\right)$ and the deformation-induced stress field resulting from unclamping $\left(\sigma_{xx,\;u}\right)$, which can be expressed by Equation (1) [5,13,57,58], where the term $\left(\sigma_{xx,\;i}\right)$ can be assumed or measured experimentally; $\left(\sigma_{xx,\;u}\right)$ can be obtained by DIC-monitored unclamping. Equation (1) is schematically represented in Figure 3.

(1) $$\sigma_{xx,\;res}\left(y\right)=\sigma_{xx,\;i}+\sigma_{xx,\;u}$$

#### 2.3.2. Assumptions for Initial Stress Field

Based on experimental observations reported in the literature [5,13,57,58,59], assumptions made to simplify residual stresses analysis using the DIC technique are as follows:

- There is no or close-to-zero deflection in the rigidly clamped component during the WAAM deposition process. Once unclamped, the bending moment acting on the cross-section is zero [5].
- The deposited wall initially contains tensile residual stresses due to the restriction to shrinkage deformation during cooling down using rigid clamps. The longitudinal residual stress field that is initially present in a clamped component can be approximated by a constant (or uniform) stress over the height of the wall [5,58]. The magnitude of this stress depends on the thermal properties, and for steel, this can be as high as the material’s yield strength at room temperature [58]. This assumption also provides a safety margin, as equalizing to yield strength represents the worst-case scenario [5,13,59].
- In the substrate, the extent of the plastic zone is negligible in the clamped state and residual stresses are entirely compressive, with a non-continuous transition at the interface wall/substrate to balance the process-induced tensile residual stresses in the deposited wall [5].

#### 2.3.3. DIC-Monitored Unclamping

**Figure 4:**
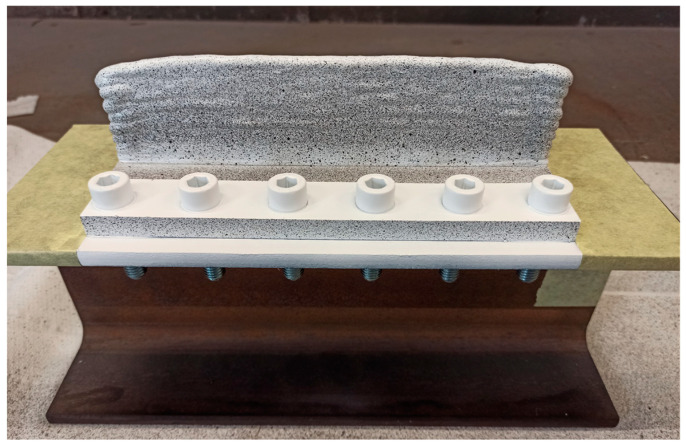
A specimen (wall—48 mm height, clamped on an H profile) with speckle patterns on the wall and substrate front.

The underlying concept of DIC involves comparing images of a component taken before and after deformation. For the DIC-aided unclamping, the visualised surfaces on which deformation information was to be known were specially treated in advance. All specimens were first cleaned with P180 sandpaper and remaining dirt/dust particles on the surface were removed with a pneumatic air jet. Next, white paint (MoTip Matt White spray) of about 6 layers was applied on one side of the wall specimens and substrate fronts. Afterwards, black random speckles (MoTip Matt Black spray) were applied onto the white surface to achieve a surface with a high-contrast composition of ~50% white and ~50% black. A speckled specimen clamped to an H profile is shown in Figure 4.

**Figure 5:**
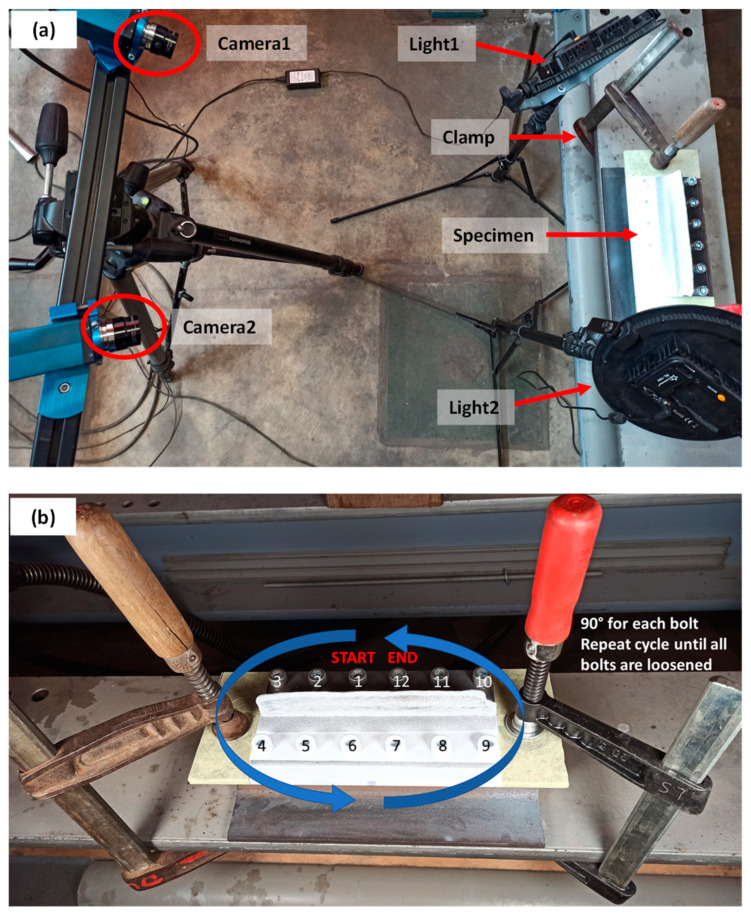
(**a**) Set-up for DIC-monitored unclamping, (**b**) Procedure followed to unscrew the bolts gradually.

To perform the DIC measurements, a set of two cameras, two lenses, a tripod, standing lights and a computer with appropriate DIC software were used at the Soete Laboratory of Ghent University. The two cameras were mounted such that the two regions of interest could be captured, namely the wall front and the substrate front. Figure 5a shows the DIC-monitored unclamping setup. The lenses used were 25 mm in diameter, and the cameras had a resolution of 5 MPx (2452 px by 2054 px). The depth of field was implemented accordingly such that both regions of interest could be captured with relatively low uncertainty, which was accomplished by adjusting the focus of the lenses. The aperture was set at 4 for all measurements, and the angle between both cameras (stereo-angle) was 20–25°. The stereo-plane was set at an angle with the ground of approximately 30°; this was to capture the wall front without the bolt heads blocking the view. Before the actual measurements, the cameras were calibrated by using a predefined grid (12 × 9–5 mm) around three perpendicular axes. During the tests, images were captured by the cameras using the Vic-Snap 8 software, and the correlation software Vic-3D 7 was used to process captured digital images [60].

Finally, to capture the deformation-induced strains during unclamping, the component was gradually unbolted and pictures were taken between each 90° (approx.) unscrewing of a bolt. In between each unscrewing, three images were taken. The unscrewing started with bolt 1 and followed the arrows shown in Figure 5b to complete one cycle. During the DIC-monitored unclamping process, two hand clamps were used to clamp the part-substrate-H profile configuration to a rigid and vibration-free foundation. The strain field ($\varepsilon_{xx}$ and $\varepsilon_{yy}$) obtained from DIC-monitored unclamping was converted to stresses in the longitudinal direction $\left(\sigma_{xx,u}\right)$ using Equation (2) for plane stress conditions at a free surface [61], where υ (0.3) represents the Poisson’s ratio and E (210 GPa) is the elastic modulus. The final residual stress is calculated by Equation (1).

(2) $$\sigma_{xx,u}=\frac{E}{\left(1-\upsilon^{2}\right)}\left(\varepsilon_{xx}+\upsilon \varepsilon_{yy}\right)$$

### 2.4. Validation and Verification of DIC Results

Once the unclamping stresses in all specimens were measured using DIC-monitored unclamping, established residual stress measurement methods (contour method and XRD) were used to validate the DIC results. Additionally, an analytical model was used for the verification of experimental results.

**Figure 6:**
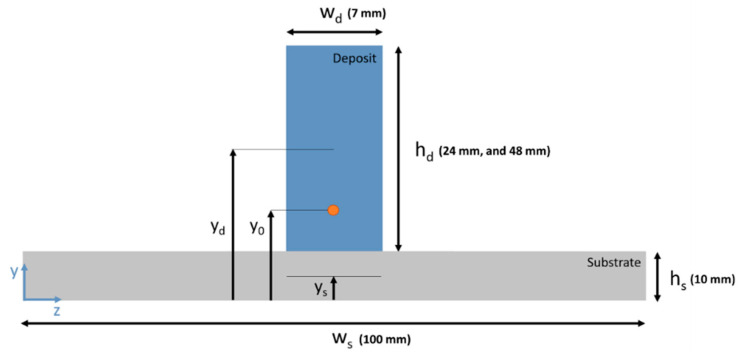
Schematic of the cross-section of a WAAM part (wall deposited on a substrate) [13].

(a) Contour method: The contour method is a destructive residual stress measurement technique based on stress relaxation [38]. A part containing residual stress is cut into two halves, and the stress component normal to the cut surface is measured. At Coventry University, contour measurements were carried out on two specimens, one each from 24 mm and 48 mm wall height (specimen IDs 24-S2 and 48-S1). Samples were cut on a Fanuc Robocut α-C600i wire electro-discharge machine (Fanuc, Yamanashi, Japan). A brass wire of 0.25 mm diameter was used. Symmetric and rigid clamps were used while cutting. The samples were first cut through the deposit (starting from the top end of the deposit) and finally through the substrate. The cutting speed for all samples was ~0.5 mm/min in the deposit and less in the substrate. The surface displacement profile of the cut surfaces of the samples was measured with a Zeiss Contura g2 coordinate measuring machine (CMM) (Zeiss, Rugby, UK) using a 3 mm diameter touch-trigger probe. The distance from the perimeter and between the individual measurement points of the sample surface was set as 0.2 mm.
  The displacement data of the cut surfaces of each sample were post-processed using Matlab analysis routines for data aligning, cleaning, flattening and smoothing. The data smoothing of all samples was conducted using a cubic spline with 3 mm knot spacing. A finite element (FE) model of one cut half of the samples was built with 8-node brick elements (C3D8R) of the Abaqus software. A mesh of approx. 0.5 mm size was used on the cut surface. Constraints were applied to the model to avoid rigid body motion. Linear elastic FE analysis with the following material properties was performed to calculate the residual stresses present in the samples before cutting: E = 210 GPa, and υ = 0.30.
(b) X-ray diffraction (XRD): XRD measurements were carried out using a mobile Stresstech X3000 (Stresstech Ltd, Vaajakoski, Finland) with Cr-radiation (30 kV and 8mA) at Sirris. Residual stresses were measured along the centre of a 48 mm wall (48-S2), from the top of the wall to its mid-height. Two sets of measurements were performed on the same locations, one without any surface treatment and one after electropolishing (180–200 µm below the surface). All measurements were performed in the omega-mode using a 3 mm diameter collimator at the Bragg diffraction angle (2θ) of 156.4° for Fe (211) reflection and with a wavelength (λ) of 2.291 Å at an angle of 0° direction (i.e., along the longitudinal or deposition direction of the wall). For each measurement point, there were seven tilt or inclination angles (ψ) varying between 0° and 39.8° using 3 s exposure time. For peak fitting, the Pseudo Voigt method was used for all measurements.
(c) Analytical method: The stress field caused during unclamping could be calculated using the principles of solid mechanics, as proposed by Hönnige et al. [13]. Stress caused by unclamping $\left(\sigma_{xx,u}\right)$ was calculated using Equation (3), and then residual stresses $\left(\sigma_{xx,\;res}\left(y\right)\right)$ were calculated using Equation (1) assuming the initial stress field in deposited walls $\left({\sigma_{xx,\;i}}_{d}\right)$ equalled the tensile yield strength of the material, i.e., 502 MPa. Figure 6 schematically represents the cross-section of the substrate and deposited wall, indicating geometric parameters, where subscript “*d*” stands for deposit and “*s*” for substrate. The parameter “*w*” represents the width, either of deposit or substrate, whilst parameter “*h*” represents the height. The y-axis lies in the direction of the wall height. The location of the neutral axes of deposit and substrate is characterized by their y-coordinate with respect to the bottom of the substrate.
  (3) $$\sigma_{xx,u}=\frac{My}{I_{zz}}$$
  where bending moment $M=\frac{1}{2}F\left(h_{s}+h_{d}\right)$, $F={\sigma_{xx,\;i}}_{d}w_{d}h_{d}={\sigma_{xx,\;i}}_{s}w_{s}h_{s}$, and $I_{zz}$ is the moment of inertia.

## 3. Result and Discussion

### 3.1. Strain Field Captured during DIC-Monitored Unclamping

**Figure 7:**
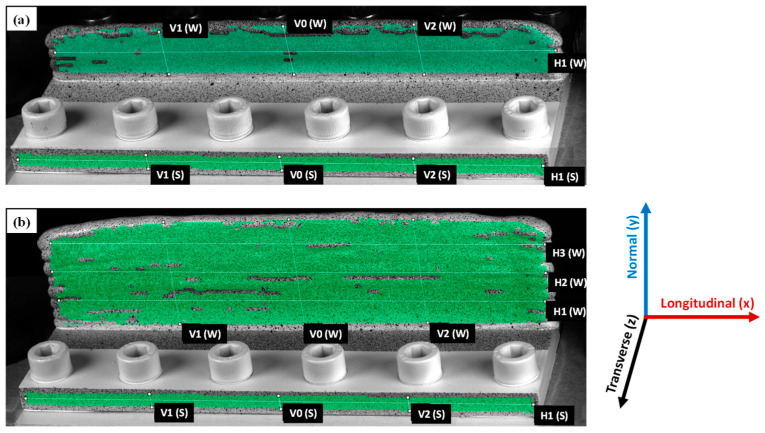
Regions of interest and location of the lines to extract results for comparisons: (**a**) 24 mm wall, (**b**) 48 mm wall.

The regions of interest that the DIC software was able to capture and process (on the substrate fronts and WAAM wall fronts) are the coloured regions as shown in Figure 7a,b. Additionally, lines were drawn across the region of interest, and strains along those lines were compared for the different specimens. Lines V0(W,S) always represent the vertical centre lines, whilst lines V1(W,S) and V2(W,S) lie vertically towards the left and right from the centre of the wall (W) and substrate (S) front. Lines H1(W,S) are the horizontal lines at the substrate (S) and wall (W) fronts. For the walls of 48 mm height, additional horizontal lines H2(W) and H3(W) were drawn along the wall length. As can be seen in Figure 7a,b, the regions of interest on the wall front have some discontinuity, which is due to poor speckle pattern contrasts or shadows caused by the waviness of the deposited wall.

**Figure 8:**
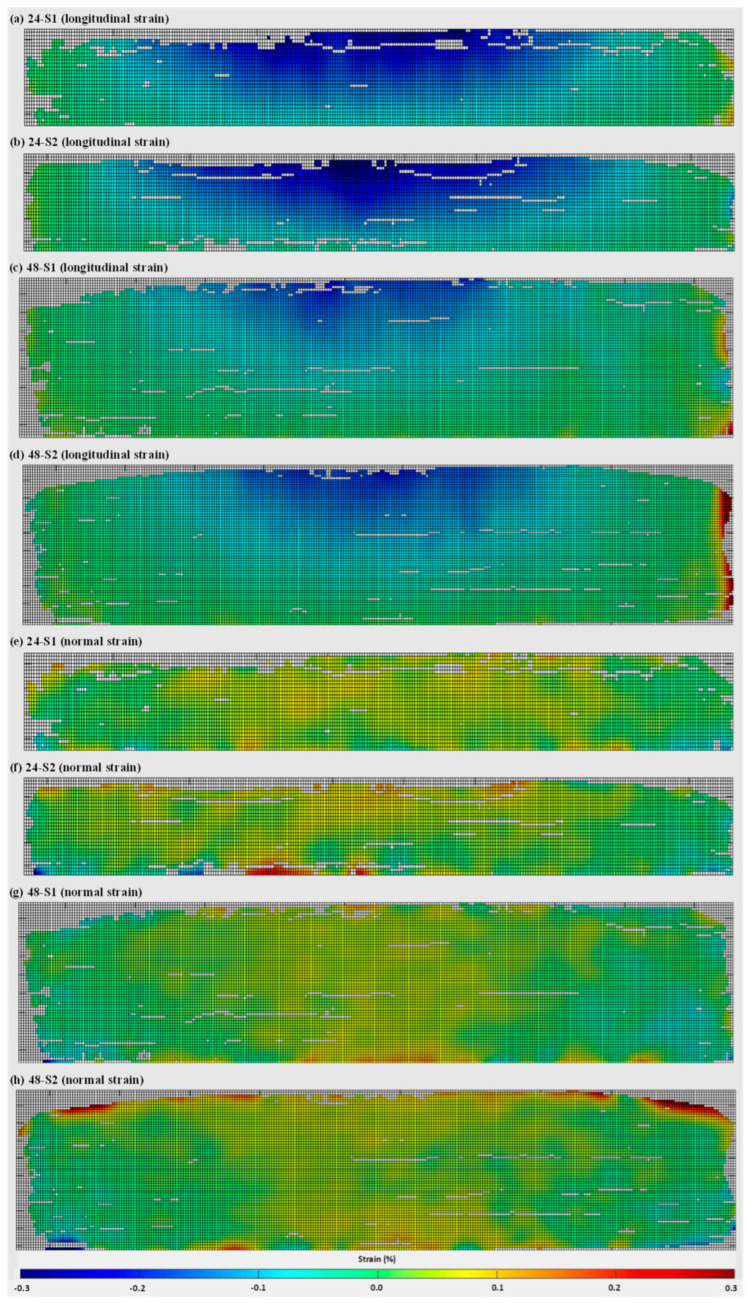
Two-dimensional strain distributions on the WAAM wall fronts captured during DIC-aided unclamping: (**a**–**d**) longitudinal strains, (**e**–**h**) normal strains. (**a**,**b**,**e**,**f**) represent the 24 mm wall, and (**c**,**d**,**g**,**h**) represent the 48 mm wall; S1 and S2 represent two repeating tests.

The 2D full-field strain distributions (normal and longitudinal directions) on the wall fronts of 24 mm and 48 mm specimens are presented in Figure 8. As can be seen from Figure 8a–d, compressive longitudinal strains were determined with a semi-elliptical shape from the top centre of both wall heights, as the walls bent during unclamping due to shrinkage deformation. Gradually, the nature of strain distribution changes from compressive to tensile towards the wall edges (left, and right), and near the interface as the substrate obstructs the wall from deforming. The distributions of normal strains are presented in Figure 8e–h and found to be more uniform and lower in magnitudes when compared to longitudinal strains, although there are some spikes close to the interface and near the wall edges (which might be due to the so-called edge effect).

**Figure 9:**
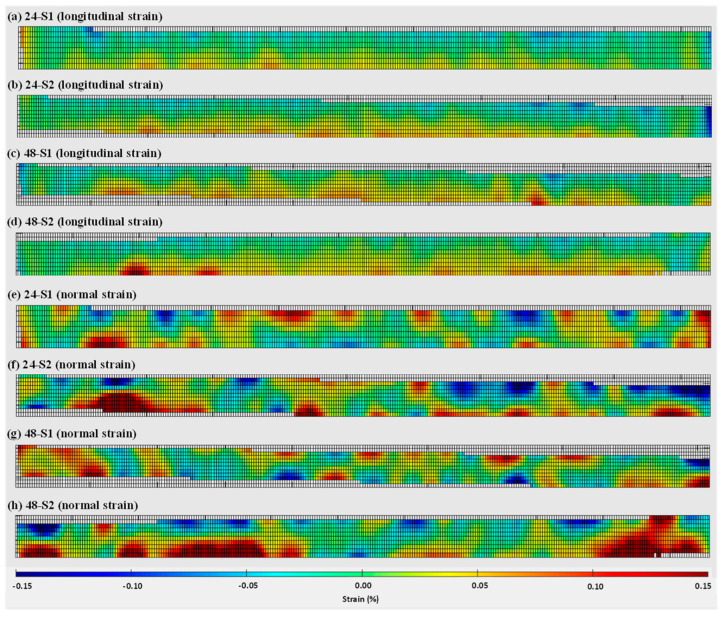
Two-dimensional strain distributions on the substrate fronts captured during DIC-aided unclamping: (**a**–**d**) longitudinal strains, (**e**–**h**) normal strains. (**a**,**b**,**e**,**f**) represent the 24 mm wall, and (**c**,**d**,**g**,**h**) represent the 48 mm walls; S1 and S2 represent two repeating tests.

Figure 9 represents the strain distribution on the substrate fronts for all specimens being investigated. As the wall–substrate configuration bent during unclamping, DIC captured compressive longitudinal strains occurring in the top half of the substrate front, whilst tensile longitudinal strains occurred in the bottom half (Figure 9a–d). When compared to the wall fronts, the substrate fronts possess a lower magnitude of longitudinal compressive strains. Regarding the distribution of normal strains, clusters of compressive and tensile strains were observed with no clear trend (Figure 9e–h), which might be due to the presence of the fastening bolts. Notably, when compared to the longitudinal strains on the substrate front, the normal strains reach much higher values.

**Figure 10:**
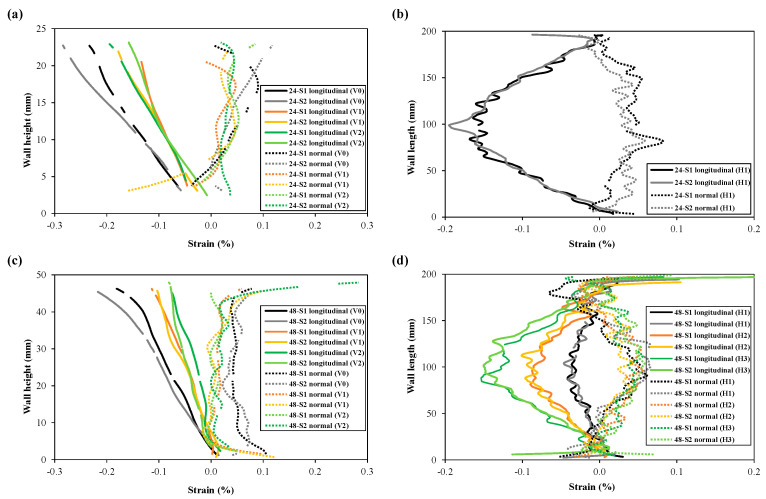
Comparison of longitudinal and normal strains on the wall fronts extracted from different regions (lines) of the WAAM walls: (**a**,**b**) 24 mm walls, (**c**,**d**) 48 mm walls; (**a**,**c**) represents strain along the wall height and (**b**,**d**) strain along the wall or substrate length. (Line numbers can be found in Figure 7).

**Figure 11:**
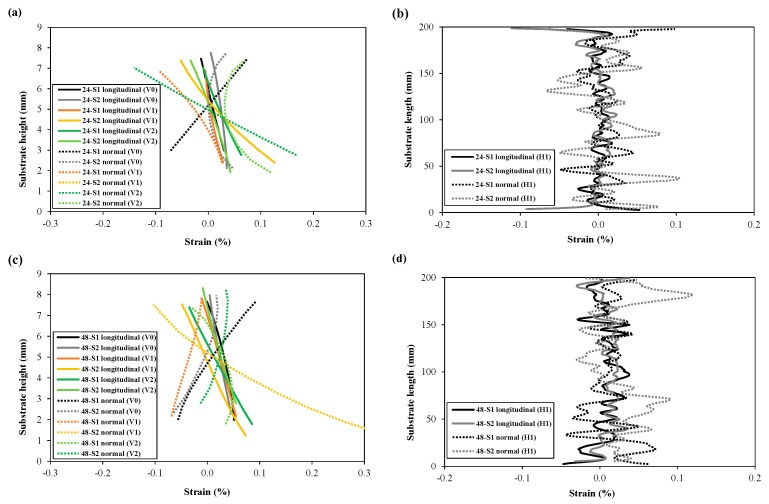
Comparison of longitudinal and normal strains on the substrate fronts extracted from different regions (lines) with 24 mm walls (**a**,**b**), and 48 mm walls (**c**,**d**); (**a**,**c**) represents strain along the wall height and (**b**,**d**) strain along the wall or substrate length. (Line numbers can be found in Figure 7).

The longitudinal and normal strains resulting from unclamping were extracted from the different lines (shown in Figure 7) and plotted for comparison in Figure 10a–d (wall fronts) and Figure 11a–d (substrate fronts). As can be seen from Figure 10a,c, the highest compressive strains were observed at the top of the wall for both wall heights, having the highest gradient along the centre line (V0) and gradually approaching close-to-zero values near the substrate. Strain plots along the vertical lines V1 and V2 also show symmetry in strain distribution at equal distances from the centre line V0. Symmetry in strain distribution can also be observed in the plot of the longitudinal stresses along the horizontal line H1 (for the 24 mm wall) and H1, H2, and H3 (for the 48 mm wall). Plots from horizontal lines show compressive strains at the wall centre, highest at the wall top (H3 for 48 mm wall), lowest at the bottom (H1 for 48 mm wall), and gradually becoming close-to-zero values towards the wall edges.

The normal strain values seem to be slightly positive and fluctuate close-to-zero along the vertical lines (V0, V1 and V2). The magnitude of normal strains is much smaller compared to the longitudinal strains on the wall. Moreover, there is no clear difference in normal strain distribution among different vertical lines. The normal strain profile along the horizontal line H1 shows a trend of increase in normal strains at the wall mid-length.

Figure 11a–d shows the longitudinal strain (line plots) for the substrate fronts of specimens with both wall heights. Longitudinal strain distributions along the vertical lines (V0, V1, and V2) show a linear trend, compressive on the substrate top to tensile towards the substrate bottom due to the bending deformation, as shown in Figure 11a,c. When compared to the walls, the symmetry in strain distribution towards either side of the centre line (V0) is less obvious, which is also noticeable in the horizontal line H1 plots in Figure 11b,d. Ups and downs in longitudinal strain distribution along H1 could be due to the presence of bolts in the substrate, and their movements during unclamping might have influenced the strain evolution, although the bolt clamping and unclamping procedures for each specimen were performed as consistently as possible. Likewise, normal strains on the substrate fronts show random patterns for all line extractions both in the vertical and horizontal directions. Another explanation might be that because the substrate’s region of interest was very small, the influence of the edge effect was much larger compared to the wall fronts.

The line plots also demonstrate repeatability in strain measurements during DIC-monitored unclamping for identical specimens (S1 and S2 for each of the two wall heights), particularly the longitudinal strain component for wall fronts. Small deviations might be caused by natural scatter in residual stress fields or minor location differences of the lines across the wall fronts, as the drawing of these lines was a manual operation. The repeatability in longitudinal strain at the substrate surface seems to be less compared to the wall front, and therefore, substrate results are considered less reliable. Regarding normal strains measured on the substrate, there is no repeatability in DIC captured strains. Overall, longitudinal strains (i.e., strains along the direction of deposition) were found to be more critical than normal strains.

### 3.2. Residual Stresses Evaluated Using the DIC-Based Approach

**Figure 12:**
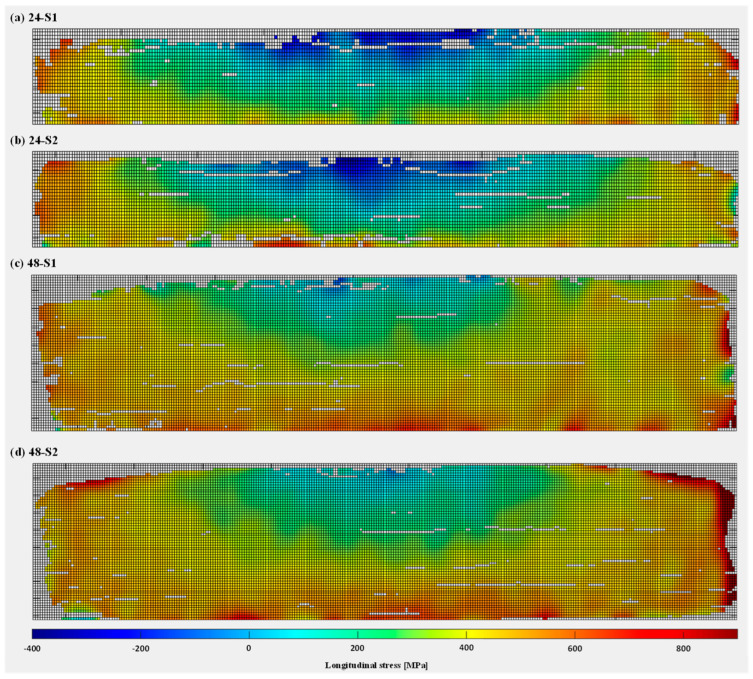
Longitudinal residual stresses in the WAAM wall fronts with heights 24 mm (**a**,**b**) and 48 mm (**c**,**d**): S1 and S2 are repeating tests for each wall height.

**Figure 13:**
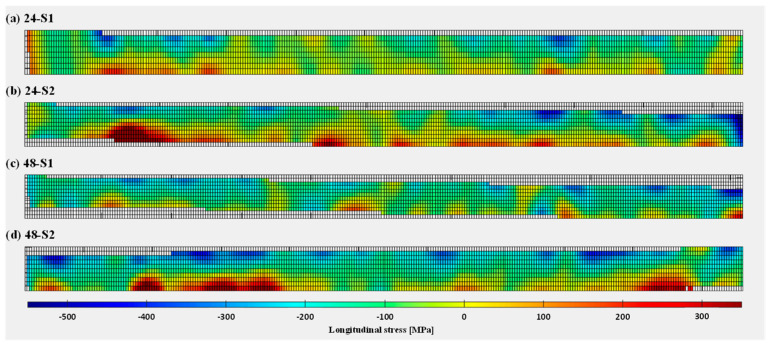
Longitudinal residual stresses in the substrate fronts with wall heights 24 mm (**a**,**b**) and 48 mm (**c**,**d**): S1 and S2 are repeating tests for each wall height.

The final longitudinal residual stresses calculated from DIC results are presented in Figure 12 (wall front) and Figure 13 (substrate front), using Equation (1). Similar to the strain fields obtained after DIC-aided unclamping (Section 3.1), residual stresses were found to be compressive at the top centre of the WAAM walls, gradually changing to tensile towards the substrate as well as on the right and left edges of the walls. In other words, unclamping causes distortion and redistribution of the stresses in such a way that there is a uniform gradient from compressive residual stresses of a semi-elliptical pattern (at the top centre of walls) to tensile residual stresses (towards the substrate and wall side edges). The compressive and tensile stresses along the height of the walls can be explained by the fact that the highest layers are deposited later on. The bottom layers have already cooled down and shrunk a bit when a new hot layer is added on top. As this new layer cools down, shrinkage is restrained by the underlying layer, causing the part to distort [62]. Residual stress results for wall fronts also demonstrated the repeatability in residual stress measurements for both wall heights (specimens S1 and S2). Regarding the longitudinal stresses in the substrate fronts, the lower half of the substrate contains tensile stress and the upper half contains compressive stress. The tensile and compressive stresses are, however, present in the form of clusters rather than a continuous distribution along substrate length. Therefore, the reliability of the substrate results may be questioned. When comparing the evolution of longitudinal residual stresses for the 24 mm and 48 mm walls, it was observed that the 24 mm wall specimens possess higher magnitude compressive residual stresses at the top of the wall, because the shorter walls deform more due to their lower cross-sectional stiffness. On the other hand, the 48 mm wall specimens reach higher tensile stresses in the wall close to the substrate due to the higher restraint from the substrate.

### 3.3. Validation of DIC Results: Comparison with XRD, Contour Method, and Analytical Prediction

**Figure 14:**
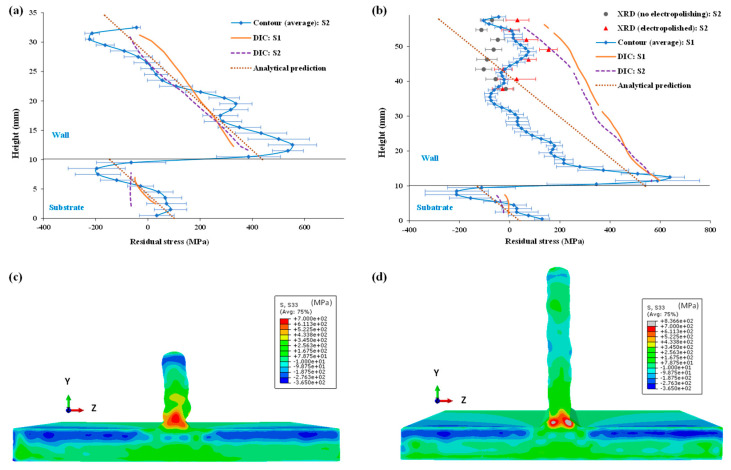
Comparison of longitudinal residual stresses along specimen centrelines measured using proposed DIC-based approach, contour method, and XRD and predicted by analytical model: (**a**) 24 mm wall, (**b**) 48 mm wall; two-dimensional residual stress maps produced by the contour method (longitudinal stress component): specimens (**c**) 24 mm wall, (**d**) 48 mm wall.

To validate residual stress results, DIC measurements (extracted along vertical centreline V0 for specimens) were compared with two established residual stress measurement techniques and an analytical model. Figure 14a shows a comparison with the contour method and analytical prediction for the 24 mm walls. Figure 14b shows a comparison among DIC, contour method, XRD, and analytical prediction for the 48 mm walls. It can be observed that the DIC results for the 24 mm specimens agree quite well with the contour method measurements (average values in thickness direction, z) and analytical model, for both wall and substrate. However, for the 48 mm wall, it can be seen from the contour method results that the stress distribution becomes more complicated and did not follow a linear trend (i.e., compressive at the top to tensile at the bottom of the wall). XRD measurements performed on the top half of the wall (especially, in electropolished condition) also correspond well with the contour method results. The non-linearity on the 48 mm walls could be due to large shrinkage stress, as residual stresses in WAAM structures originate from welding or restraint stresses due to plastic strain mismatch followed by shrinkage stresses (due to elastic strain mismatch) once deposition reaches a certain height. On the other hand, the DIC analysis and analytical model are based on linear elastic stress assumptions and also assume uniform yield stress distributions for the clamped specimens. This could be a reason for the deviation of DIC based results from the contour method, and XRD results. Additionally, the theoretical model describes longitudinal stresses in a perfect T-profile, whilst DIC-monitored unclamping took place for the wall-substrate configuration with a curved top, especially the corners, i.e., lower height near the end of the weld lines. Furthermore, the deviation between DIC and XRD method is also possible because the DIC method is based on strains at the macroscopic level, whilst the XRD method is sensitive to microstructural heterogeneity. Nevertheless, DIC results at the top and bottom of the wall agree well with contour method results and the analytical model predictions.

**Figure 15:**
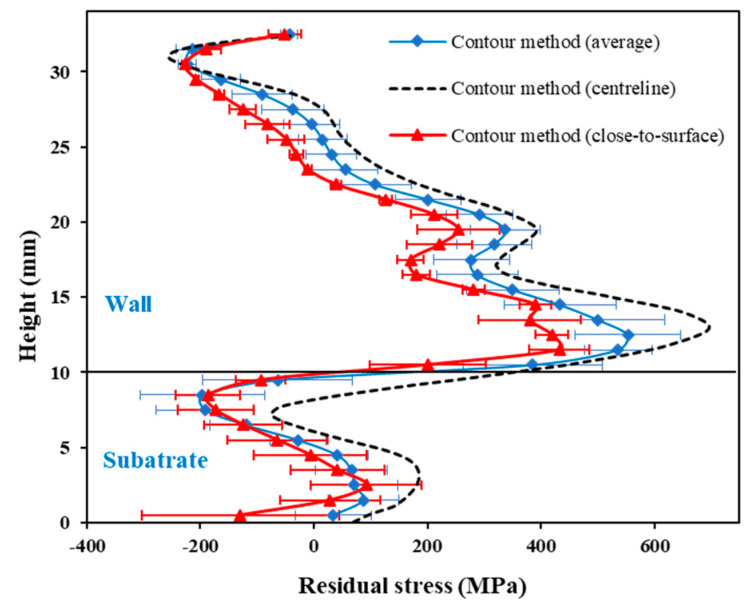
Comparison of residual stresses among “average” (along z-direction), “centreline”, and “close-to-surface” values (average of two nodes from the surface), measured via contour method for a 24 mm wall.

It is arguable that the DIC captured strains and resulting residual stresses are only representative of the surface strain/stress state. However, from the contour method measurements, it was observed that there was no significant difference in longitudinal residual stress along the thickness direction (z), except in the substrate just below the interface. Figure 15 shows a comparison among longitudinal residual stress extracted from contour measurements: average values in the thickness direction (z), centreline values, and close-to-surface (average of two nodes from the wall or substrate edges). Therefore, it can be concluded that the strains and residual stresses measured using the proposed DIC-based approach not only demonstrate surface strain or stresses but are also representative of the strain or stress state along the thickness direction (z) of the slender walls.

## 4. Conclusions

A digital image correlation (DIC) based methodology was developed for measuring full-field residual stresses in wire and arc additive manufactured (WAAM) components. To demonstrate this, a case study was performed for WAAM walls with two different heights (24 mm and 48 mm) deposited using AWS ER70S-6 steel alloy wire. Residual stresses measured using the DIC-based method were validated using the contour method and X-ray diffraction (XRD) and verified using an analytical model. The conclusions from this study can be summarised as follows:

- Strain measurements (particularly in the longitudinal direction) obtained from DIC-monitored unclamping demonstrated good repeatability for both wall heights. Repeatability in measurements on the substrate fronts can be questioned due to its small region of interest.
- The proposed DIC-based method allowed capturing of full-field residual stresses for the entire WAAM wall front, showing compressive longitudinal residual stresses at the top centre region of the wall, gradually changing to tensile stresses toward the interface and edges.
- For the 24 mm walls, higher compressive stresses in the longitudinal direction were observed at the top centre of the wall. On the other hand, the magnitude of tensile residual stresses near the interface was higher for the 48 mm walls.
- Residual stress measured using the proposed DIC-based approach demonstrated a good agreement with the results from established stress measurement techniques (contour method, XRD) and analytical predictions, especially for the shorter walls with 24 mm height.

## Figures and Tables

**Table 1:** Established experimental residual stress measurement techniques: accuracy, depth of measurement, and stress state [55].

Measurement Technique *		Accuracy (MPa)	Depth (mm)	Stress State
Semi-destructive	Centre-hole drilling	10–30	2	Allows bi-axial residual stress measurements
	Deep hole drilling	10–30	750	Bi-axial measurement; tri-axial is possible, but with extra difficulty and reduced accuracy
	Ring coring	10–30	5 (25 with core removal)	Bi-axial measurements
	Sachs boring	10–45	>100	Bi-axial measurements
Destructive	Slitting	10–30	>100	Uni-axial measurements
	Contour method	Depends on cutting, measuring, smoothing, and filtering methods.	Specimen thickness	Uni-axial measurements
Non-destructive	X-ray diffraction	7–20	0.01–0.02 (standard) 1–1.5 (with electro-polishing)	Bi-axial measurements
	Synchrotron diffraction	10–30	20 (steel) 100 (aluminium)	Tri-axial measurements
	Neutron diffraction	10–30	60 (steel) 100 (aluminium)	Tri-axial measurements
	Ultrasound	Not reported	150	Tri-axial measurements

* DIC-based techniques are not included in this table.

**Table 2:** Chemical content (% weight) of key alloying elements for the feedstock wire and substrate material.

Alloying Elements	C	Mn	Si	P	S	V	Cu	Cr	Ni	Mo	Al	Nb	Ti
ER70S-6 wire	0.08	1.70	0.85	0.007	0.007	0.05	0.20	0.05	0.05	0.05	-	-	-
S355 substrate	0.12	1.50	0.50	0.025	0.020	0.20	-	-	-	-	0.015	0.09	0.15

**Table 3:** Key process parameters used to manufacture specimens.

	Shielding Gas	Welding Mode	Gas Flow (l/min)	Welding Speed (m/min)	Wire Feed Speed AVG (m/min)	Stick-Out (mm)	Current AVG (A)	Voltage AVG (V)	Interlayer Temperature (°C)
Layer 1	Ar + 18% CO_2_	CMT	15	0.35	7.8	17	205	13.2	80–100
Layer 2, 3, …17	Ar + 18% CO_2_	CMT	15	0.35	6.5	17	188	12.6	80–100

**Table 4:** Specimen details used for evaluation of residual stresses using the DIC technique (unit: mm).

Specimen Types	Wall Dimension (Length × Height × Width)	Number of Layers	Number of Specimens	Specimen IDs	Measurement Methods
Wall—24	200 × 24 × 7	8	2	24-S1	DIC
				24-S2	DIC, contour method
Wall—48	200 × 48 × 7	17	2	48-S1	DIC, contour method
				48-S2	DIC, XRD

## Data Availability

We declare that the data underlying this article are original and are fully available and obtainable from the corresponding author.
